# The dung beetle microbiome complements host metabolism and nutrition

**DOI:** 10.1128/msystems.01172-25

**Published:** 2025-10-28

**Authors:** Joshua A. Jones, Armin P. Moczek, Irene L. G. Newton

**Affiliations:** 1 Department of Biology, Indiana University 1772, Bloomington, Indiana, USA; University of Connecticut, Storrs, Connecticut, USA

**Keywords:** *Onthophagus*, symbiosis, microbiome, developmental biology, arthropods, dung beetle

## Abstract

Many multicellular organisms rely on communities of microbial organisms to properly benefit from their diets, for instance, by assisting in the breakdown of complex polysaccharides, the synthesis of essential resources, detoxification, or even preventing putrefaction. Dung beetles commonly rely on herbivore dung as their main source of nutrition, a diet rich in recalcitrant, hard-to-digest plant polysaccharides yet poor in essential amino acids, which animals typically cannot synthesize on their own. The work presented here investigates the potential role of the host-associated microbial community in allowing these insects to thrive on their nutrient-poor diet. Specifically, we investigated whether the microbiota of the bull-headed dung beetle, *Onthophagus taurus*, may be capable of synthesizing amino acids and breaking down complex plant polysaccharides. To do so, we functionally annotated genes within metagenomically assembled genomes (MAGs) obtained via shotgun-metagenomic sequencing. The annotation of these MAGs revealed that bacteria found in association with *O. taurus* possess the metabolic potential necessary to bridge the gap between host metabolic needs and the limitations imposed by their diet. Specifically, *O. taurus* microbiota contain amino acid biosynthesis pathways and genes encoding cellulases and xylanases, both of which are absent in the beetle genome. Further, multiple functionally relevant bacterial taxa identified here have also been observed in other studies across diverse dung beetle species, possibly suggesting a conserved pool of dung beetle symbionts and metabolic functions.

**IMPORTANCE:**

Host-symbiont interactions allow animals to take advantage of incomplete and/or challenging diets and niches. The work presented here aims to identify the physiological and metabolic means by which host-associated microbial species shape the ecology of one of the most speciose genera in the animal kingdom: dung beetles in the genus *Onthophagus*. Both larva and adult stages of most *Onthophagus* rely on herbivore dung, a diet rich in recalcitrant, hard-to-digest plant polysaccharides yet poor in essential amino acids, which animals typically cannot synthesize on their own. To utilize such a challenging diet, *Onthophagus* vertically transmits a maternally derived microbial community which supports normative development in immature individuals and maintenance and reproduction in adults. Taken together, *Onthophagus’* extraordinary diversity, complex ecology, and varied relationship with their microbial associates make them an ideal system to investigate mechanisms and diversification of host-diet-microbiome interactions.

## INTRODUCTION

Animals often rely heavily on key symbionts within their microbiome to exist within their ecological niche (1–3). Depriving these animals of their microbes often renders them unable to use their focal food resource as their symbionts provide key metabolic pathways (1, 4, 5). For example, insects such as the rhinoceros beetles, termites, and leafcutter ants lack the ability to reliably break down the complex plant polysaccharides in their diets (6–8). Instead, these insects harbor bacterial or fungal symbionts with the metabolic potential to produce diverse enzymes to break down these resources and provide simpler components to the host. Conversely, many animals consume diets that are rich in simple carbohydrates yet poor in essential nutrients that the animals cannot make. For example, aphids, bees, and stink bugs all consume diets rich in sucrose, fructose, and glucose but poor in essential amino acids. However, in each case, microbial symbionts are capable of synthesizing essential amino acids to the benefit of their host (9–11). The importance of these interactions to animal resource use suggests that host-symbiont interactions are essential to understanding animal ecology and evolution.

Dung beetles, which specialize in the dung of other animals, are another clade of animals reliant on a challenging diet. As such, Onthophagine beetles often use herbivore dung as both a dietary and reproductive resource (12), which presents further difficulty because of the abundance of tough-to-digest plant materials and relative lack of essential nutrients in the dung itself (13, 14). Despite this, dung beetles are extraordinarily species-rich, with the genus *Onthophagus* alone accounting for an estimated 2,500 extant species (15). Recent work suggests that onthophagine dung beetles may owe a portion of their evolutionary success to a community of heritable and functionally significant microbes. Inhibiting the inheritance of these microbes results in prolonged developmental time and decreased adult size (16), suggesting that these beetles are reliant on their microbiome for normative development. This, in light of their difficult diet, has fueled the hypothesis that dung beetles rely on the metabolic pathways encoded within their microbial associates to efficiently utilize and complement their diets. To date, the only data supporting this hypothesis rely on either 16S rRNA-based functional predictions (17, 18), which, however, often miss functional differences within taxa (19), or the metabolic activity of isolated microbes (20), which focuses on compounds that are likely digestible by host genomes (21). Here, we set out to construct a metagenome of the symbionts of the bull-headed dung beetle, *Onthophagus taurus,* to test the hypothesis that the dung beetle microbiome encodes metabolic capabilities that empower dung beetles to utilize an otherwise hard-to-digest and incomplete diet.

To accomplish this, we sequenced the bacterial community from the *O. taurus* larval gut, the adult midgut, and the pedestal (a fecal pellet left by ovipositing mothers), which assists in the passage of microbes across generations (16, 22, 23). These reads were assembled into individual metagenomically assembled genomes (MAGs) and annotated to functionally and taxonomically characterize the dung beetle microbiome. Finally, the metabolic potential of the microbiome was compared across three onthophagine dung beetles (*O. taurus*, *Onthophagus sagittarius*, and *Digitonthophagus gazella*) to determine if functional deficits present within the host genomes are complemented by the genes present in the microbiome. Our results suggest that the dung beetle microbiome has the potential to compensate for deficiencies in the host genome with respect to both complex polysaccharide breakdown and essential amino acid synthesis. Further, taxonomic identifications provide evidence that Bacteroidales and Pseudomonadota may be particularly important in fulfilling these roles in *O. taurus*.

## MATERIALS AND METHODS

### Sample preparation, sequencing, quality control, and assembly

Details on the specific methodologies used to produce samples, sequences, and MAGs are described in the companion *Microbiology Resource Announcements* article (24). In brief, five libraries were produced by pooling samples across five sample types: larval foregut, midgut, and hindguts, adult midguts, and pedestals. For larval samples, the foregut, midgut, and hindguts were differentiated based on an apparent cuticle layer present on the foregut and hindguts but not the midgut (Fig. S1). In samples where this was not immediately apparent, these sections were differentiated by a slight narrowing between the foregut and midgut and by the point where the malpighian tubules connect to the gut, thereby marking the boundary between the midgut and hindgut. For adult samples, the midgut was the entire portion of gut from the inception of the thorax until the attachment point of the Malpighian tubules. Importantly, these samples were enriched for bacteria (following methods modified from (25) decreasing the representation of DNA from the host and other eukaryotes. A total of 32 MAGs were produced, and 16 were determined to be >90% complete (using CheckM (26) and uploaded to NCBI in BioProject PRJNA1117517 (see Table S1 for SRA accession numbers). The assemblies for the remaining 16 MAGs were uploaded to the DRYAD repository. Details on MAG assembly quality, content, identification, and the total proportion of assembly reads binning within the MAG are summarized in Table S1.

### Characterization of microbial communities

Community composition was determined after initial quality control steps (removing host-associated DNA sequences and trimming low-quality reads) and before assembly. Reads were processed on KBase (27) using Kaiju v1.9.0 (28), in greedy mode, and the Kaiju databases: RefSeq Complete Genomes (protein sequences from completely assembled bacterial, archaeal, and viral genomes from NCBI RefSeq updated 23 March 2022 and fungus protein sequences from a representative set of fungal genomes updated 23 March 2022). Kaiju output contained the relative and total abundance of each taxon; no normalization or transformation was conducted. The resulting compositional data were transferred to R v4.2.2 (29) for figure production and to determine which taxa were shared across samples. The Kaiju outputs of each sample are deposited into the DRYAD repository.

### Determining the amino acid synthesis pathway completion

Metabolic potentials for *O. taurus*, *O. sagittarius*, and *D. gazella* were all determined using DNA sequences and gene predictions from Davidson & Moczek 2024 (30). To avoid associating genes from any potential microbial contamination within the beetle genome assemblies, analysis was limited to the definitive chromosomes. These include: chr1, chr2, chr3, chr5, Schr6, chr7, chr8, chr10, chr11, ScKx7SY_15, and ScKx7SY_16 for *O. taurus*, Scaffold_1, Scaffold_2, Scaffold_3, Scaffold_4, Scaffold_5, Scaffold_6, Scaffold_7, Scaffold_8, Scaffold_9, and Scaffold_10 for *O. sagittarius*, and ScIV947_1, ScIV947_2, ScIV947_3, ScIV947_5, ScIV947_6, ScIV947_7, ScIV947_9, ScIV947_12, ScIV947_14, and ScIV947_38 for *D. gazella*. The prediction of beetle amino acid synthesis pathways was determined by annotating the beetle genomes with the KEGG reference database (31) and searching for amino acid synthesis genes in each genome. A pathway was considered complete if the enzyme catalyzing each reaction between pyruvate and that amino acid was present (32, 33). The amino acid synthesis potential of the MAGs was determined with Gapseq (34), with default parameters, which produced predictions for the completeness of amino acid biosynthesis modules. If a module was incomplete, any module requiring that one as a prerequisite was also considered incomplete.

### Annotating carbohydrate-active enzymes

The carbohydrate breakdown potential was quantified by using the KEGG annotations from the beetles and the RASTtk (35–37) annotations from the MAGs. RASTtk annotations for all 32 MAGs are deposited into the DRYAD repository. Direct breakdown potential for major dung components (cellulose, cellobiose, xylan, and xylose) was quantified by counting related genes throughout these annotations, specifically cellulose breakdown genes were typically associated with EC 3.2.1.4, while xylan breakdown genes were either EC 3.2.1.8 (endo-1,3-beta-xylanases), or EC 3.2.1.37 (xylan 1,4-beta-xylosidases). To determine further carbohydrate breakdown potential, CAZymes (Carbohydrate-Active enZYmes) within the beetle genomes were identified using the dbCAN HMM database v12.0 and instructions found here (https://bcb.unl.edu/dbCAN2/download/Databases/dbCAN-old@UGA/) (38). In short, HMMER v3.4 (39) (hmmer.org) was used to determine which genes within each beetle genome were annotated in the CAZyme database before a parser script was used to assign those genes into CAZyme families. In parallel, annotated MAGs were input into the dbCAN2 app (38, 40, 41) (v1.9.1), on KBase, to produce similar annotations based on CAZyme families. We focused on the number of genes assigned to glycoside hydrolase (GH) families, quantifying their abundance across each beetle genome and MAG.

## RESULTS

### Assembly results and statistics

Initial sequencing and quality control resulted in a total of 54,481,286 reads across our five libraries. These reads were assembled into a total of 26,749 contigs, which were binned into 32 MAGs. A total of 28.4% reads mapped to these 32 MAGs or the host genome (20.9% and 7.53%, respectively), suggesting we captured only a subset of the total microbial diversity present in these environments within these MAGs. For this manuscript, we focus on raw reads to estimate community composition, contigs to estimate overall metabolic potential, and the 16 nearly complete MAGs, with a completion score above 90% in CheckM (26), alongside the 16 less complete MAGs, with completion between 44.0% and 89.8% for more specific functional predictions and annotations.

### Bacterial and fungal composition

**Fig 1:**
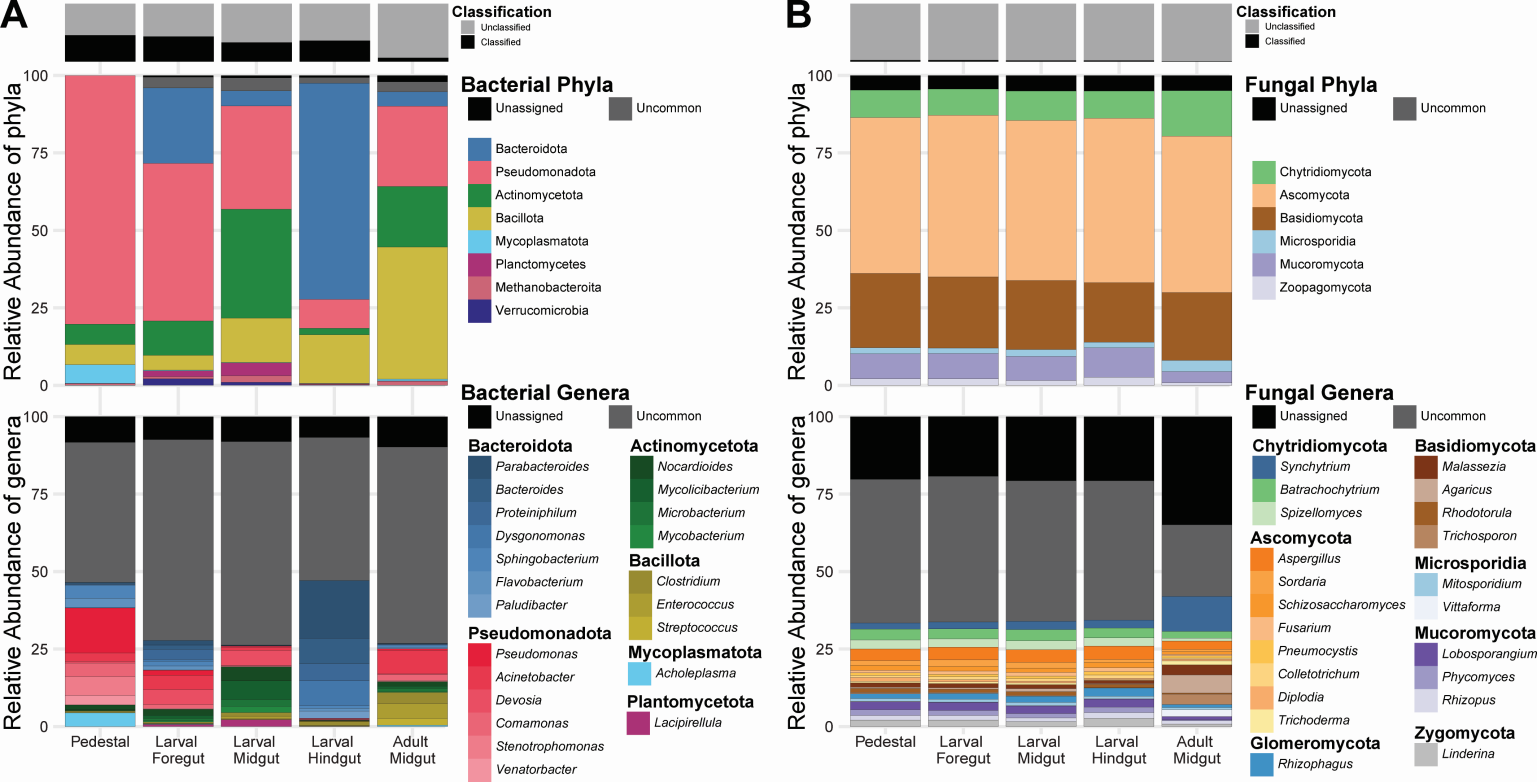
Relative abundance of bacteria and fungi across sample types. (**A**) shows the relative abundance of the most common bacterial phyla (middle) and genera (bottom) across samples, and (**B**) shows this data for fungi. Bar plots on top show the total percentage of quality-controlled reads from the sample that could be assigned as bacterial (**A**) or fungal (**B**). “Uncommon” taxa were those that could be classified yet did not represent more than 2% or 1% relative abundance (for bacteria and fungi, respectively) within any sample. Finally, taxa that couldn’t be identified to the phyla or genera level were combined to form the “Unassigned” category.

To gain insights into any potential community differences across these sample types, we assessed the bacterial and fungal communities using unassembled reads characterized with Kaiju. The Greedy mode of Kaiju translates DNA into proteins and breaks reads into fragments before identifying a read based on the database sequence with the highest possible similarity score, allowing for substitutions (28). This results in a higher rate of identification of genera underrepresented in the database and allows for novel genera to be identified to the family level (28). Kaiju was able to classify 6–54% of reads as bacterial and 0.6–2.6% as fungal across samples, with the adult midgut having the lowest classification rate and the pedestal having the highest for both bacteria and fungi (Fig. 1; Fig. S2 and S3). The much lower classification rate of fungi was likely the result of our sample processing (i.e., enriching for bacterial reads to decrease host reads likely also decreased fungal reads) and not a representation of their differential abundance. Across all samples, Kaiju identified 1,720 bacterial families, 65% of which were present in all samples (Fig. S4). The fungal communities identified were less diverse overall, with 222 fungal families being identified, 85% of which were present in all samples. No single bacterial genus dominated any sample, but the most abundant genera were all Pseudomonadota or Bacteroidota (Fig. 1A).

### Deficits in host amino acid synthesis pathways are present in microbial genomes.

**Fig 2:**
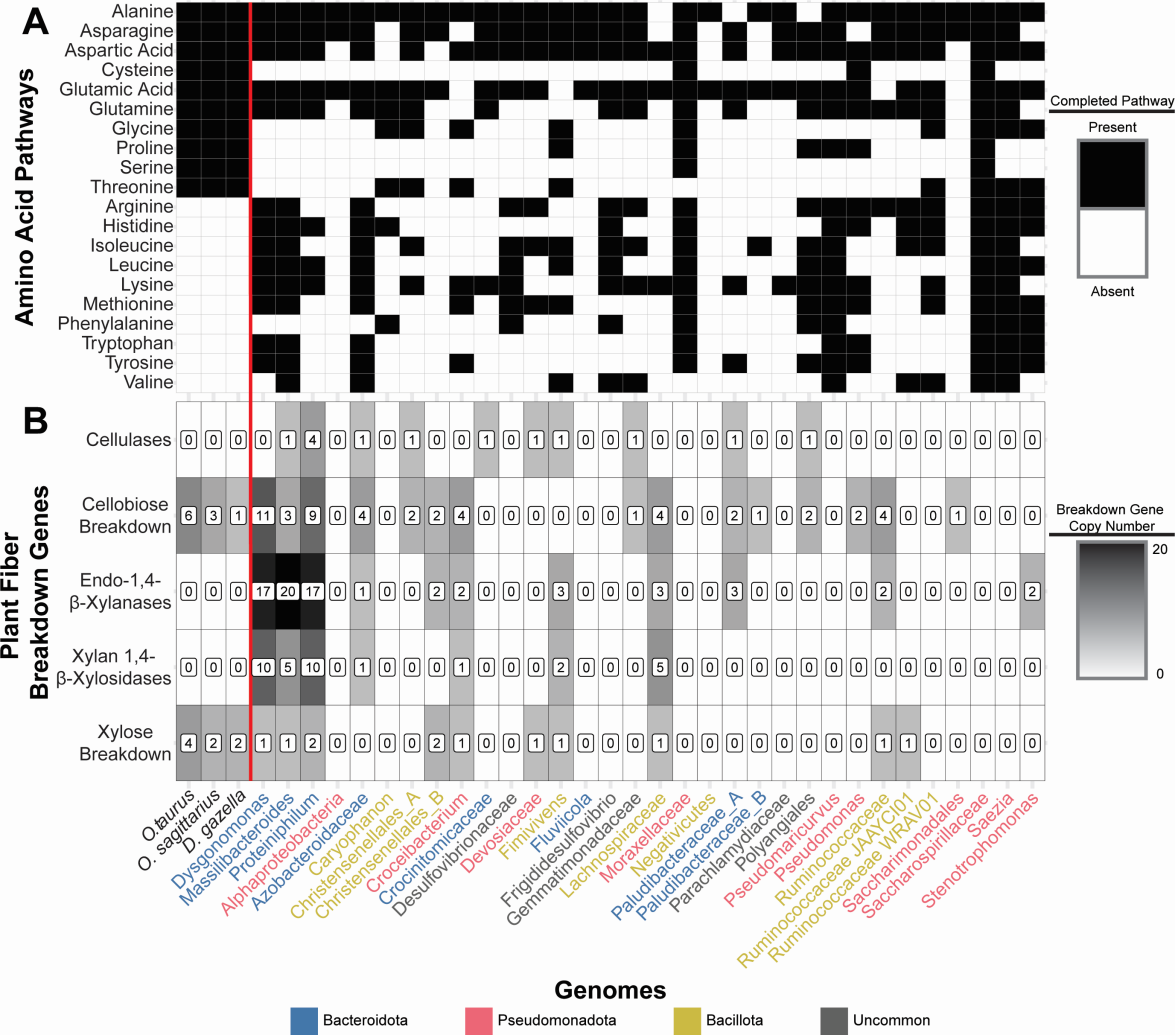
Metabolic potentials of beetles and gut bacteria. (**A**) Presence/absence of completed amino acid pathways. Each beetle genome contains the genes to synthesize 10 of the 20 essential amino acids, while metabolic modeling predicts that microbes present in *O. taurus* can synthesize all 20. (**B**) Abundance of putative cellulose and xylan breakdown genes. Bacteria found in *O. taurus* contain genes to break down cellulose, xylan, and their simpler components, while beetle genomes only contain the genes to break down the simple components. Colored MAG names represent the phyla each MAG was classified into, colors match in Fig. 1A, and gray names are those from phyla that were in the “uncommon” group in Fig. 1A.

As herbivore dung may be poor in essential amino acids (13, 14, 42), we identified complete amino acid synthesis pathways in the host and microbial genomes to ascertain if deficits in host metabolism could be supplemented through biosynthesis by microbes. All three dung beetle species (*O. taurus*, *O. sagittarius*, and *D. gazella*) appear to possess the same amino acid synthesis potential as other arthropods (43), retaining the genes to encode enzymes necessary to synthesize only 10 of 20 amino acids (Fig. 2A). The microbial genes represented throughout the MAGs have the potential to encode enzymes for synthesizing all 20 common amino acids. Saccharospirillaceae was the only MAG that contained the genes necessary to synthesize all 20 amino acids, with the next highest potentials (18 amino acids) found in the Moraxellaceae and *Saezia* (26). The remaining MAGs varied in potential, encoding genes able to synthesize between two and sixteen amino acids. Thus, the onthophagine gut microbiome appears to contain multiple copies of amino acid synthesis pathways not found in host genomes.

### Bacterial genomes contain complex polysaccharide breakdown genes absent from host genomes.

The dung diet comprises complex polysaccharides from plant cell walls, often recalcitrant to animal digestion (44, 45), though some animal genomes do encode key plant cell wall breakdown genes (46–48). To understand how the beetle may overcome this obstacle, we identified genes encoding enzymes that may break down these complex polysaccharides or their simpler components. Neither the *O. taurus*, *O. sagittarius*, nor *D. gazella* genomes contained genes homologous to any cellulase or xylan-breakdown genes (Fig. 2B). Yet all three genomes contain homologous genes to enzymes which break down the simpler components of those polysaccharides, cellobiose (*n* = 6, 3, and 1, respectively) and xylan (*n* = 4, 2, and 2, respectively). In contrast, dbCAN2 identified 13 putative cellulases and 106 putative xylan-breakdown genes across the 32 MAGs. The majority of the xylan-breakdown genes (*n* = 72) were endo-1,4-beta-xylanases, with the remaining (*n* = 34) being xylan 1,4-beta-xylosidases. Although the MAGs encoded more enzymes associated with xylan breakdown than cellulose breakdown, we identified more genes associated with the degradation of cellobiose (*n* = 52) than xylose (*n* = 12). Across all MAGs, *Proteiniphilum* encoded the largest number of genes across these categories with 31 total cellulose or xylan breakdown genes, followed by *Dysgonomonas* with 27 and *Massilibacteroides* with 26.

**Fig 3:**
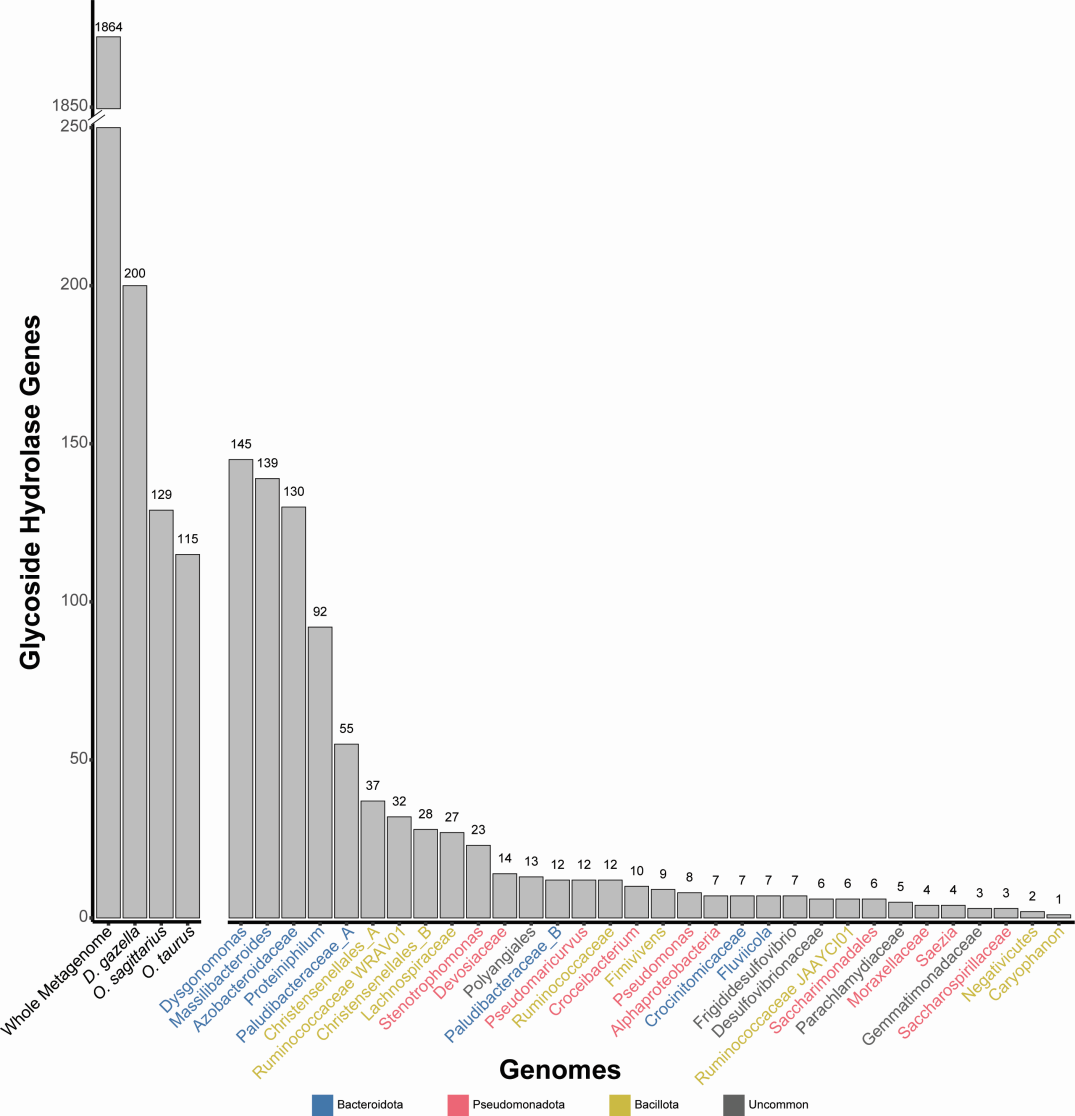
Abundance of putative GH genes within the whole metagenome, individual beetle genomes, and individual MAGs. The *D. gazella* genome contains a far greater number of potential GH genes than those of *O. sagittarius* or *O. taurus* as well as the individual MAGs, which have abundances similar to *O. sagittarius* and *O. taurus*. The counts within the beetle genomes, as well as the individual MAGs, are all dwarfed in comparison to the culmination of potential GH genes throughout the entire metagenome. Colored MAG names represent the phyla each MAG was classified into, colors match Fig. 1A, and gray names are those from phyla that were in the “uncommon” group in Fig. 1A.

To get an idea of the broader polysaccharide breakdown potential, we also quantified the total number of predicted GH genes across both host genomes and MAGs (Fig. 3). GHs are a broad group of enzymes defined by their potential to hydrolyze glycosidic bonds. This family of genes includes those capable of breaking down cellulose, cellobiose, xylan, and xylose, as discussed above, in addition to many other carbon sources. By quantifying GHs, we were able to achieve an estimation of the potential for these organisms to digest a broader variety of complex polysaccharides, which they may encounter. We found that *D. gazella* possesses the highest GH count (200), whereas *O. sagittarius* and *O. taurus* featured more similar counts at 129 and 115, respectively. However, these values are dwarfed by the GH abundance across all assembled contigs (*n* = 1,864) and are comparable to the highest values within individual MAGs (*Dysgonomonas* = 145, *Massilibacteroides* = 139, and *Azobacteroidaceae* = 130). More generally, these results suggest that the microbial community may expand the ability for the dung beetles to derive nutrition from their diet by encoding an abundance of polysaccharide-utilization genes.

## DISCUSSION

Many animals that consume complex or nutrient-poor diets rely on microbial symbionts for digestion or nutrient synthesis (1, 3, 5, 44, 48). Onthophagini beetles obligately consume herbivore dung as a diet throughout development and into adulthood (22). Herbivore dung is a challenging diet because it is mostly composed of tough plant materials which animals often can’t digest (6, 7, 42). Additionally, essential amino acids, which animals must consume from their diet, are also rare in herbivore dung (13, 42). Together, this makes dung a particularly difficult food source and suggests that dung beetles may require their microbes to both digest and synthesize essential resources from this diet. Here, we combined analysis of the metabolic potential of three onthophagini beetle genomes (*O. taurus*, *O. sagittarius*, *D. gazella*) with that derived from shotgun-metagenomic sequencing of *O. taurus* gut sections and pedestals. By comparing these data sets, we validated expectations of metabolic deficits within the host genomes while simultaneously identifying candidate microbial symbionts to supplement these deficits.

### Evidence for diverging microbial communities across gut samples

Characterizations of the bacterial and fungal communities derived from different gut sections and host developmental stages revealed several interesting patterns. Notably, the fungal community structure was more stable between sample types than that of the bacterial community, with the majority of fungal species found across all samples. This result, obtained in this study on *Onthophagus* beetles, contrasts with previous work in another dung beetle species showing strong differentiation in fungal communities in the gut across the life stages of the dung beetle *Catharsius molossus* (49), suggesting possible differences in host-fungal interactions across the species. Compared to fungal abundance, the relative abundance of bacterial taxa across samples was more dynamic. For example, Dysgonomonadaceae and Moraxellaceae, bacteria which have previously been shown to be abundant in numerous dung beetle genera (17, 18, 23, 49–51), exhibited pronounced differences in relative abundance depending on the sample. Specifically, Dysgonomonadaceae was found in relatively robust abundance in the larval foregut and hindgut (4.59% and 15.47%) but was rare in the pedestal and larval and adult midguts (0.33%, 0.07%, and 0.41%) while Moraxellaceae was present in the pedestal, larval foregut, and adult midgut (3.00%, 4.67%, and 7.62%), but comparatively rare in the larval midgut and hindgut (1.06%, 0.11%). The presence of *Dysgonomonas* across different dung beetle species may be indicative of a shared and conserved function in digestion. Further, the abundance of Dysgonomonadaceae, a widespread and potentially beneficial family, in the hindgut fits with previous research in *Pachnoda,* which suggests that scarab beetles may have the highest densities of beneficial microbes in the hindgut (21, 52, 53). All together, these results suggest that dung beetles likely harbor the highest densities of their beneficial symbionts within the hindgut, akin to many other insects reliant on their symbionts for digestion (6, 54–57), and that *Dysgonomonas* specifically may be a functionally relevant hindgut inhabitant. Finally, the differences we observe across samples, and previous observations of strong differences across life stages (17, 23, 49), suggest that host-derived factors likely play a major role in microbial community assembly. Future work may seek to confirm these gut section dynamics with higher replication and methods allowing for increased fungal representation.

### Evidence for reliance on microbiome members for resource digestion and synthesis

We sought to determine if onthophagini dung beetles use their symbiotic microbes for the digestion of complex polysaccharides abundant in their diet. Our results show that, like many animals, the beetles lack the genes necessary for the production of enzymes to break down either cellulose or xylan, two polysaccharides abundant in herbivore dung (13). Despite this, the beetle genomes do contain genes related to the breakdown of cellobiose and xylose, simpler components of those complex polysaccharides. Together, this indicates a gap in the metabolic potential of *Onthophagus* dung beetles to digest their diet. This gap, however, may be filled by the gut microbial community, which encodes an abundance of enzymes able to break down these complex polysaccharides. The majority of these genes are related to xylan breakdown, suggesting that the bacteria may specialize in the hemicellulose within the dung, and not the cellulose itself. Other insects feeding on cellulose-rich diets have also been shown to harbor microbial taxa specializing in the hemicellulose portions of the diet (58, 59). That said, the microbiome does also encode cellulose breakdown enzymes, including an abundance of diverse GHs, suggesting broader carbohydrate breakdown potential beyond this specialization. Interestingly, many of these xylan, cellulose, and carbohydrate breakdown genes were present within Bacteroidales, namely *Dysgonomonas*, *Massilibacteroides*, and *Proteiniphilum. Dysgonomonas*, as mentioned above, appear to be prevalent across dung beetles, having been observed in *Copris incertus*, *C. molossus*, *Euoniticellus intermedius* and *E. triangulatus*, *E. fulvus*, *O. binodis*, *O. australis*, *O. hecate*, and multiple populations of *O. taurus*, as well as within each *O. taurus* life stage (17, 18, 23, 49–51, 60, 61). The consistent occurrence of *Dysgonomonas* across dung beetles, along with the functional potential described here and in other systems (62–66), suggests that *Dysgonomonas* may be particularly important to dung beetle ecology. Overall, these results support the prediction that dung beetles likely rely on their microbiome to digest the complex polysaccharides abundant in their diet and highlight some bacteria that may be key to the maintenance of this function. Future work integrating these functions with those of the fungal portion of the microbiome may yield a clearer view of the components of the dung most influential in beetle development and microbiome assembly.

Further, our results suggest that onthophagini beetles may also rely on a functionally redundant set of symbionts to synthesize amino acids from their diet. Analysis of the beetle genomes confirmed that they lack the synthesis pathways to produce 10 of the 20 amino acids, while corresponding synthesis pathways for all 20 of these amino acids are represented within multiple MAGs. The symbionts with the highest number of complete pathways belonged to Pseudomonadota, some of which encoded enzymes able to synthesize the majority (Moraxellaceae and *Saezia*), or all (Saccharospirillaceae), of the essential amino acids. Interestingly, several Bacteroidales MAGs (*Dysgonomonas, Massilibacteroidales,* and *Azobacteroidaceae*) also contained the majority of the synthesis pathways missing from the host genomes (8, 9, and 9, respectively). Yet, MAGs assembled from Bacteroidales contained fewer total complete pathways than those assembled from Pseudomonadota, with *Dysgonomonas* containing 13, *Massilibacteroidales* containing 14, and *Azobacteroidaceae* containing 14 compared to the 20, 18, and 17 of Saccharospirillaceae, Moraxellaceae, and *Saezia,* respectively. The potential role of Pseudomonadota in dung beetle ecology may thus be important more broadly, as Moraxellaceae, in particular *Acinetobacter*, have been shown to co-occur with *Dysgonomonas* across many dung beetle species and life stages (17, 18, 23, 49–51, 61) and seem to be highly abundant within the dung they feed on (23). This supports the possibility that Moraxellaceae, and other bacteria in the dung, may be a reliable source of amino acids (13), independent of their potential to colonize within or be horizontally inherited by the host.

### Conclusion

We investigated the potential role of the microbiome in complementing the metabolic potential of dung beetles in the face of a challenging and incomplete diet. Specifically, we find that the three beetle genomes surveyed lack genes encoding enzymes necessary for breaking down complex polysaccharides or synthesizing 10 of 20 amino acids, key metabolic deficiencies given their cellulose- and xylan-rich yet amino acid-poor diet. However, the genes harbored within the gut microbiome encode the complementary metabolic potential to bridge this gap and transform dung into a resource beetles can use. Furthermore, we identify specific bacterial taxa, some of which have been found across multiple other studies in diverse dung beetle species, which may be especially important in fulfilling this function. Together, this work demonstrates how the microbiome may have scaffolded the origin and diversification of dung beetles into their unique niche. Future studies are needed to now assess the precise contributions of select microbiome members *in vivo,* and if and how microbiome diversity interacts with host diversity in development and evolution.

## Data Availability

The project discussed here has been deposited in NCBI under BioProject PRJNA1117517. Included in this are the raw sequence reads from the pedestals, larval fore-, mid-, and hindguts, and the female adult midguts (accession no.: SRR29215567, SRR29215566, SRR29215565, SRR29215564, and SRR29215563, respectively). Further, this project also includes the reads for all 16 MAGs discussed above (accession no.: SRR29455191, SRR29455190, SRR29455183, SRR29455182, SRR29455181, SRR29455180, SRR29455179, SRR29455178, SRR29455177, SRR29455176, SRR29455189, SRR29455188, SRR29455187, SRR29455186, SRR29455185, and SRR29455184). Reads for the 16 lower-quality MAGs, summary statistics for all MAGs, and the protein annotations for all MAGs are included in the DRYAD repository (https://doi.org/10.5061/dryad.4qrfj6qn8).

## SUPPLEMENTAL MATERIAL

Supplemental material msystems.01172-25-s0001.docx Figures S1 to S4 and Table S1 caption.

Table S1 msystems.01172-25-s0002.csv MAG statistics.

ASM does not own the copyrights to Supplemental Material that may be linked to, or accessed through, an article. The authors have granted ASM a non-exclusive, world-wide license to publish the Supplemental Material files. Please contact the corresponding author directly for reuse.
