# Supplementary material for: The dung beetle microbiome complements host metabolism and nutrition
Source: mSystems. 2025 Oct 28;10(11):e01172-25. doi: 10.1128/msystems.01172-25 (PMC12625706; doi:10.1128/msystems.01172-25)
Supplement: Supplemental material — Figures S1 to S4 and Table S1 caption. [file msystems.01172-25-s0001.docx]

Supplemental figures for:

The dung beetle microbiome complements host metabolism and nutrition By: JOSHUA A. JONES, ARMIN P. MOCZEK, IRENE L. G. NEWTON

Table of Contents:

| Supplemental Table 1 Legend | MAG Statistics | Page 2 |
| --- | --- | --- |
| Supplemental Figure 1 | Larval Dissection  Landmarks | Page 2 |
| Supplemental Figure 2 | Family Relative  Abundances | Page 3 |
| Supplemental Figure 3 | Absolute Abundances | Page 3 |
| Supplemental Figure 4 | Shared Taxa | Page 4 |

**Supplemental table 1.** NCBI reference identifiers, manuscript identifiers, and summary statistics for the 32 assembled MAGs.


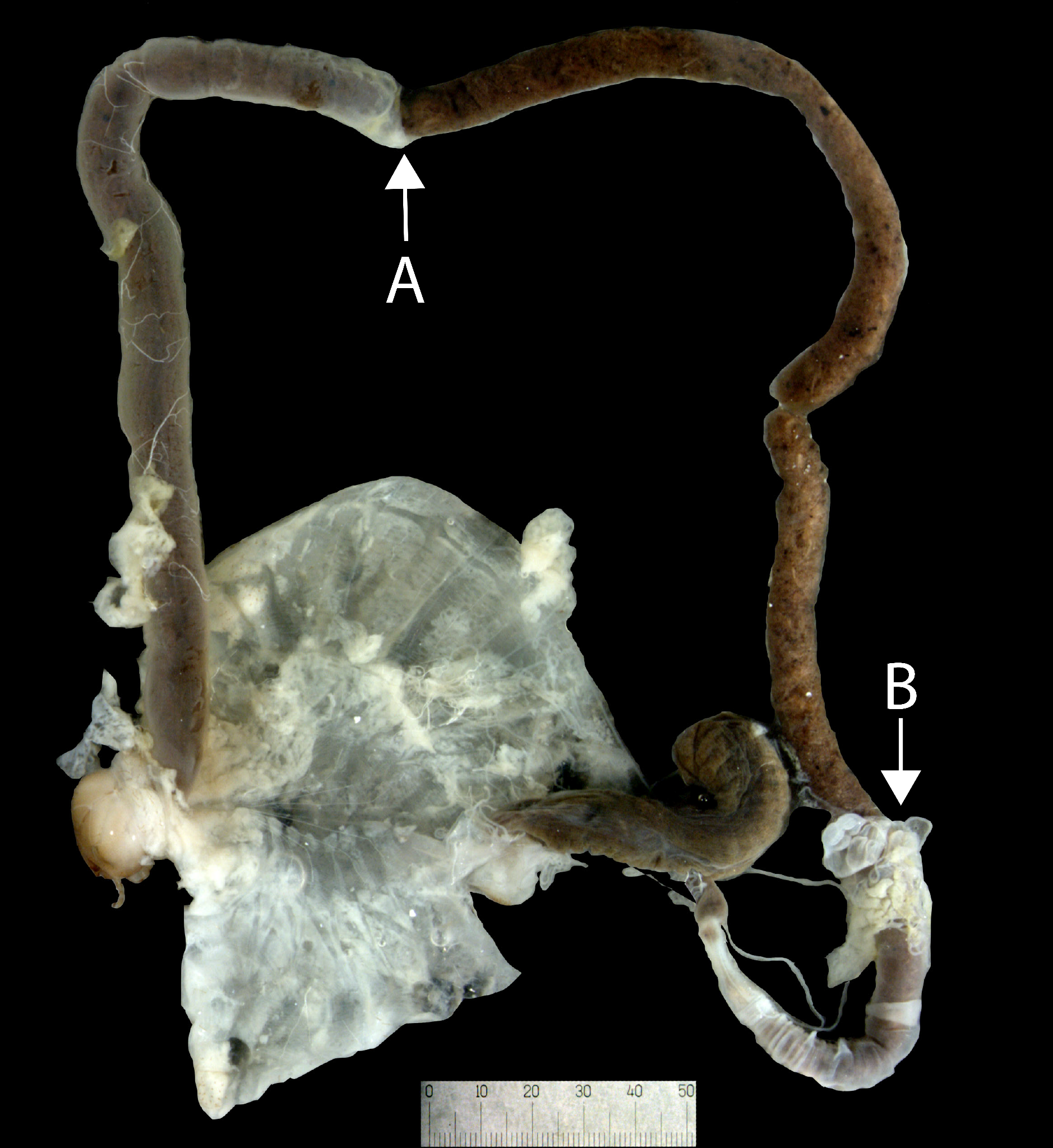


**Supplemental figure 1.** Landmarks used for larval dissections. (A) Foregut and midgut were separated at a minor narrowing in the gut where the cuticle lined foregut ends. (B) Midgut and hindgut were separated at the point where the malpighian tubules connect to the gut or where the cuticle lining of the hindgut begins.


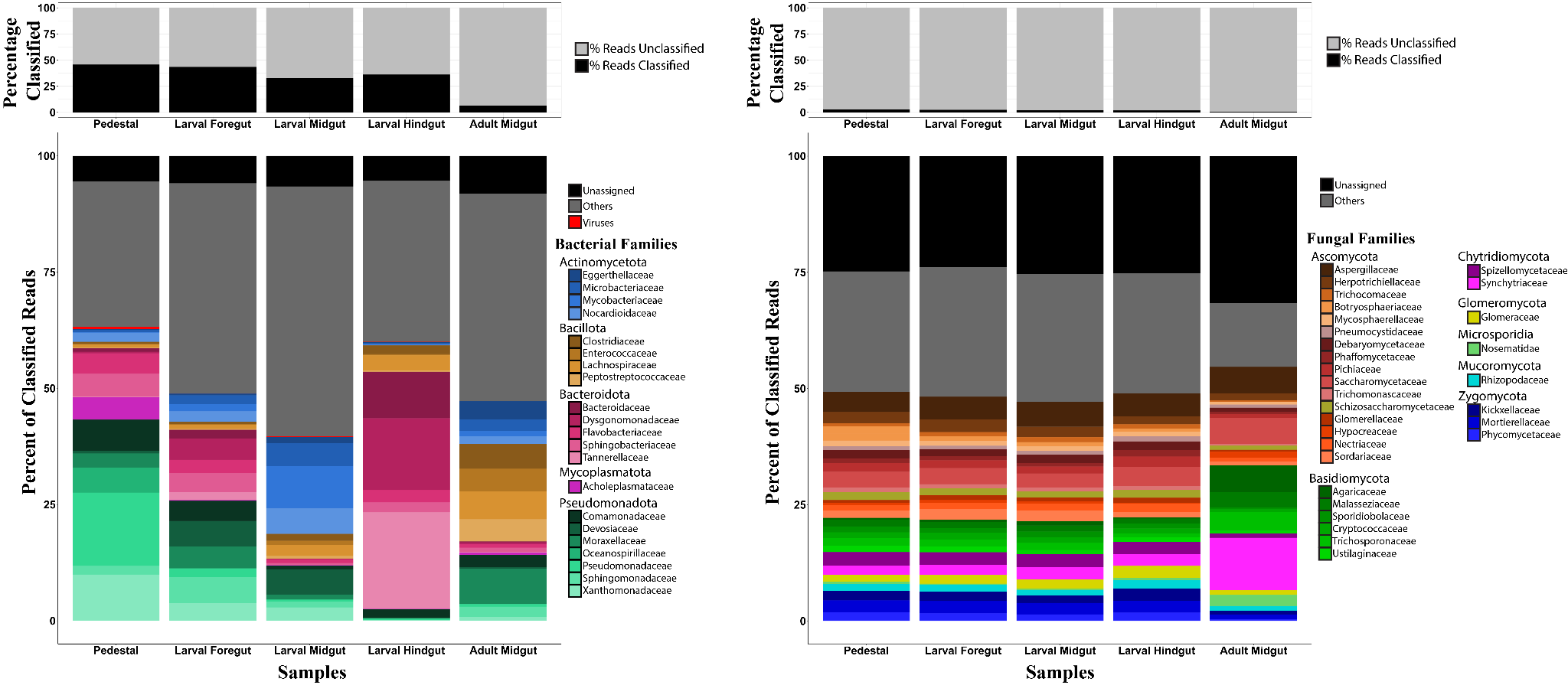


**Supplemental figure 2**: Relative abundance of bacteria and fungi across sample types. Graphs on the left show data for bacteria and graphs on the right show data for fungi. “Fraction classified”, at the top, shows the total percentage of quality-controlled reads from the sample that could be assigned to taxa in that group. “Uncommon” taxa were those which could be classified yet did not represent more than 2% or 1% relative abundance (for bacteria and fungi, respectively) within any sample. Finally, taxa that couldn’t be identified to the phyla or genera level were combined to form the “Unassigned” category..


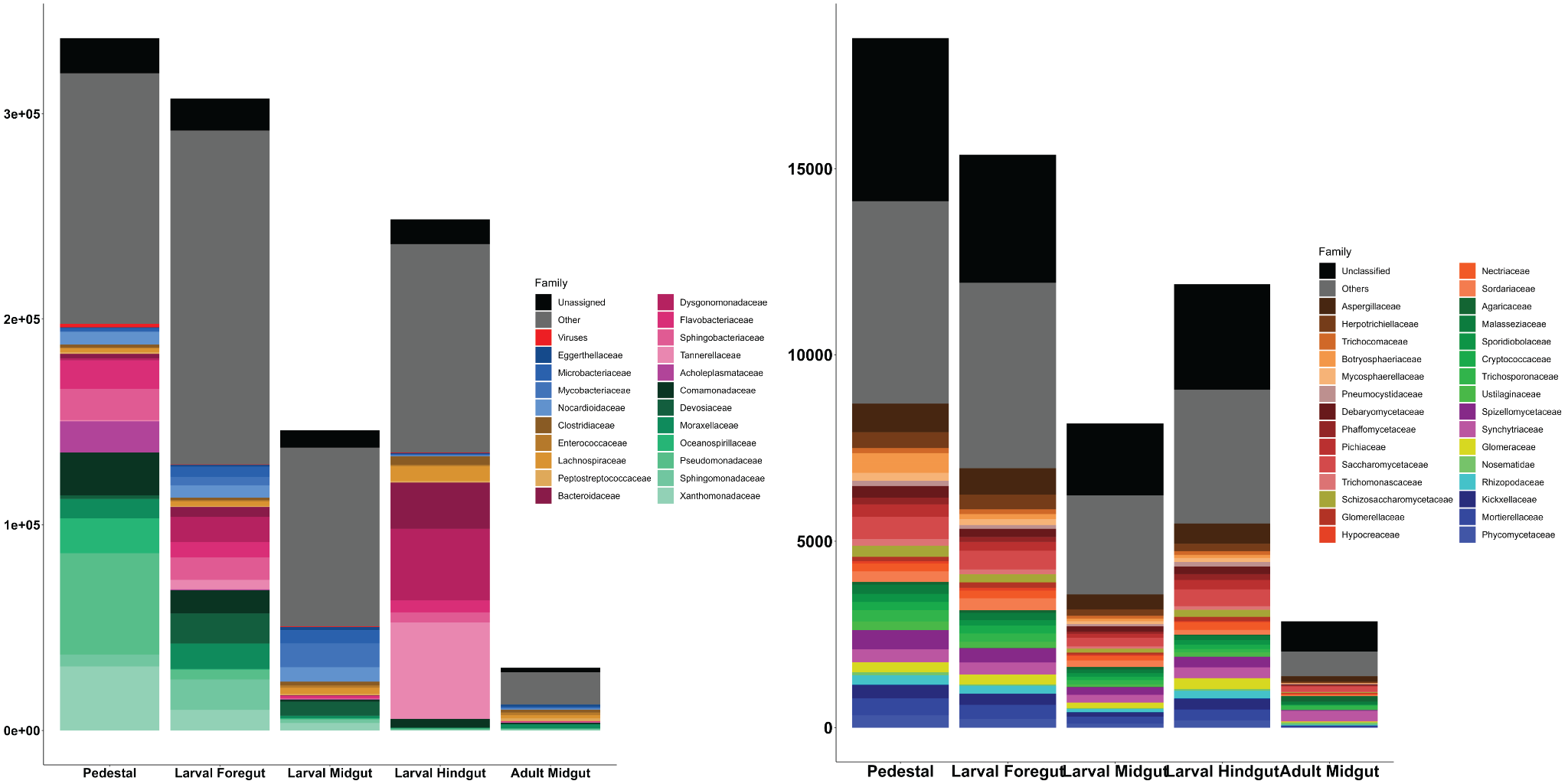


**Supplemental figure 3**: Absolute abundance of a) bacterial and b) fungal families across samples.


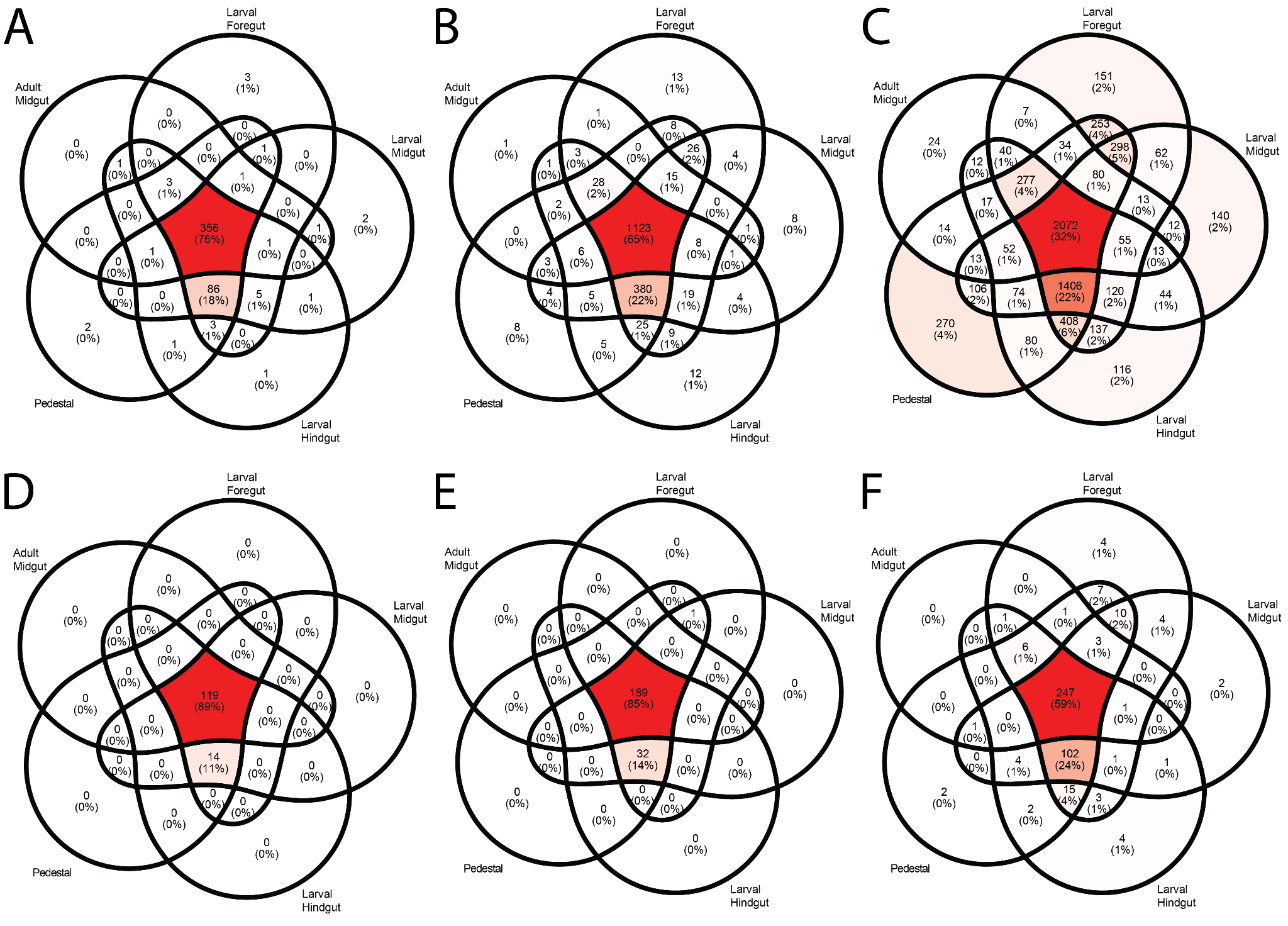


**Supplemental Figure 4**: Bacterial and fungal taxa shared across samples. A-C are bacterial families, genera, and species, respectively, and D-F are fungal families, genera, and species. The majority of taxa are found across all samples, with the exception of bacterial species. In all cases the next highest percentage of taxa are found in all samples except the female adult midgut.
